# Castor Oil-Based Epoxy Vitrimer Based on Dual Dynamic Network with Intrinsic Photothermal Self-Healing Capability

**DOI:** 10.3390/polym17070897

**Published:** 2025-03-27

**Authors:** Yingqing Shao, Haoxin Zhu, Kang Chen, Tianyi Jin, Zhiwen Wang, Zhixin Luo, Jinhui Wang, Haoyuan Sun, Shuangying Wei, Zhenhua Gao

**Affiliations:** 1 College of Material Science and Engineering, Northeast Forestry University, Harbin 150040, China; 2022111431@nefu.edu.cn (Y.S.); haoxin.zhu24@outlook.com (H.Z.); kangchen@hrbeu.edu.cn (K.C.); 15033797211@163.com (T.J.); 19163346882@163.com (Z.W.); 18755668280@163.com (Z.L.); 19945941145@163.com (J.W.); gibrilasu5@gmail.com (H.S.); 2 Engineering Research Center of Advanced Wooden Materials, Ministry of Education, Northeast Forestry University, Harbin 150040, China; 3 Key Laboratory of Bio-Based Material Science & Technology, Ministry of Education, Northeast Forestry University, Harbin 150040, China

**Keywords:** bio-based epoxy material, vitrimer, self-healing, photothermal response

## Abstract

The development of sustainable epoxy vitrimers with outstanding mechanical strength and facile self-healing capabilities are of great significance for prolonging the lifespan and enhancing the reliability of electronic devices. In this study, we present a castor oil-derived epoxy vitrimer (ASB–ECO) featuring dual dynamic networks enabled by rationally designed ester–imine bonds and an aromatic Schiff base-conjugated crosslinker architecture. This molecular design strategy effectively enhances the mechanical properties of vegetable oil-based vitrimers and endows them with controllable self-healing capabilities under photothermal conversion. The 1.0-ASB–ECO system demonstrates dynamic characteristics with an activation energy (Ea) of 37.25 kJ/mol and a topological freezing transition temperature (Tv) of 123.13 °C. The material exhibits a photothermal conversion efficiency (ηPT = 61.42%) and can achieve a self-healing rate of 100% under visible-light radiation. In addition, 1.0-ASB–ECO displays a dielectric constant (Dk) of 5.54 and a loss tangent (Df) of 0.025 at 106 Hz. This study on biomass-based epoxy vitrimers presents a novel approach to developing electronic materials, achieving a combination of high mechanical performance, sustainability, and photothermal self-healing properties.

## 1. Introduction

Epoxy resin thermosets (ERTs) are widely utilized as high-performance molding materials in various electronic applications including semiconductors [1], integrated circuits [2], and electronic components [3,4,5]. However, conventional petroleum-derived ERTs exhibit inherent limitations in sustainability due to their permanent crosslinked networks that preclude material reprocessing and circular utilization [3,6,7]. This irreversible curing behavior leads to irreversible material depletion and generates substantial non-recyclable plastic waste [8], particularly problematic in rapidly evolving electronics industries requiring frequent device upgrades [9,10,11,12]. It is essential to develop ERTs combining high mechanical properties with sustainability to extend the service life of electronic devices, support the development of a circular economy, and promote environmental sustainability [13,14,15,16,17].

Among various sustainable materials, vitrimers have gained significant attention owing to their unique combination of reversibility, self-healing capability, recyclability, reprocessability, and outstanding mechanical properties [18,19]. Consequently, vitrimers are considered promising replacements for conventional thermosetting resins, effectively integrating the beneficial characteristics of both thermoplastic and thermosetting polymers [20].

To further enhance the sustainability of these materials, current research focuses on incorporating bio-based feedstocks into vitrimer networks [21]. Epoxidized castor oil (ECO), derived from the fatty oil of the castor plant, is recognized as a promising biomass-derived epoxy precursor due to its low cost, high yield, and inherent renewability [22,23,24], making it an attractive alternative to fossil-derived raw materials [25,26,27]. Dynamic covalent adaptive networks (CANs) are one such strategy being utilized to developed bio-based epoxy vitrimers [28,29] that incorporate transesterification [30,31], imine bond exchange [19,32,33], and disulfide bond exchange [32,34]. However, a significant challenge in utilizing ECO-based epoxy thermosets lies in their typically inferior mechanical strength and relatively low glass transition temperatures (Tg) [35,36]. While vitrimer properties primarily rely on dynamic bond exchange, optimizing mechanical properties and Tg is crucial for broadening their application scope. Incorporating rigid structures into the polymer network and elevating crosslinking density serve as effective strategies to enhance the glass transition temperature (Tg) and mechanical properties of vegetable oil-based epoxy resins while preserving their inherent vitrimer characteristics [37,38].

While the plant oil-based vitrimers typically rely on bulk thermal stimulation to activate dynamic bond exchange and promote healing [30,39,40], this approach presents limitations in terms of energy consumption and potential for polymer degradation, particularly hindering localized repair. To address these challenges, photothermal stimulation offers a compelling alternative. Light energy possesses inherent advantages, including low cost, ease of accessibility, and minimal environmental impact [41,42]. Moreover, photothermal heating enables precise spatial and temporal control, minimizing energy waste and reducing the risk of thermal damage to the material [43]. Previous studies have demonstrated that certain aromatic Schiff base polymers, specifically those with highly conjugated structures, exhibit significant photothermal response characteristics due to the non-radiative relaxation of abundant delocalized π-electrons [44,45]. Organic conjugated polymers with such structural features have emerged as a promising class of photothermal materials [23], offering versatile molecular design capabilities. The photothermal effect allows for initiating the dynamic exchange of thermally responsive covalent bonds with greater spatial resolution than traditional bulk heating methods [46]. Furthermore, the light-induced temperature elevation significantly boosts polymer chain mobility [47], especially advantageous for facilitating self-healing in high glass transition temperature (Tg) polymers [43]. This photothermal incorporation thereby offers an energy-efficient strategy to achieve controlled localized self-healing in biomass-based ERTs.

In this work, we developed a photothermal-responsive bio-based epoxy vitrimer (ASB–ECO) via the heat curing reaction of epoxidized castor oil as the substrate and VAN-derived aromatic Schiff base (ASB) co-curing agent. The integrated π-conjugated system within ASB–ECO facilitates efficient photothermal conversion. The dual dynamic covalent bond network (ester–imine bonds) ensures efficient reorganization of bonds upon damage in visible light, enabling convenient recovery of mechanical properties and functional integrity. This work establishes a biomaterial paradigm combining renewable feedstocks with photothermal network control, providing a promising approach to achieving sustainability for electronic materials, potentially reducing reliance on petroleum-based polymers and minimizing electronic waste.

## 2. Materials and Methods

### 2.1. Material

Vanilin (C_8_H_8_O_3_, 99%, VAN), 4,4-Methylenedianiline (C_13_H_14_N_2_, 99%, MDA), Tris (dimethylaminomethyl)phenol (C_15_H_27_N_3_O, 99%, DMP-30), tetrahydrofuran (C_4_H_8_O, 99%, THF), methanol (CH_4_O, 99.9%, MeOH), and hydrochloric acid (36.5%, HCl) were obtained from Shanghai Macklin Biochemical Co., Ltd. (Shanghai, China); 4,4′-Dihydroxybiphenyl (C_12_H_10_O_2_, 97%, DBP) was purchased from Bide Pharmatech Co., Ltd. (Shanghai, China). Epoxidized castor oil (C_66_H_116_O_12_, 99%, ECO) was obtained from Guangzhou Yuanda New Material Co., Ltd. (Guangzhou, China). The distilled water used in this study was prepared in the laboratory.

### 2.2. Synthesis

#### 2.2.1. Synthesis of Aromatic Schiff Base Curing Agent (ASB)

Figure S1 presents a synthesis schematic of the aromatic Schiff base (ASB). VAN (12.17 g, 80 mmol) were dissolved in methanol and reacted at 65 °C under nitrogen atmosphere for 8 h. The reaction mixture was cooled to room temperature, subsequently washed with methanol, filtered, and vacuum-dried to obtain ASB as a yellow powder with a yield of ~90%.

#### 2.2.2. Synthesis of Castor Oil-Based Epoxy Resin (ASB–ECO)

**Figure 1:**
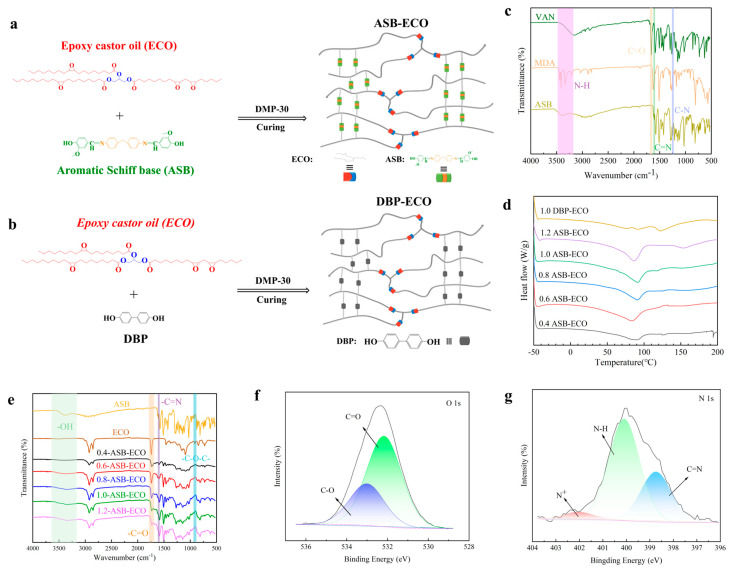
(**a**) Schematic diagram illustrating the synthesis process of ASB−ECO; (**b**) schematic diagram of the synthesis process for DBP−ECO in the control group; (**c**) FT-IR spectra of VAN, MDA, and ASB; (**d**) DSC curves of the ASB−ECO mixtures (R = 0.4, 0.6, 0.8, 1.0, and 1.2) and DBP−ECO mixtures (R = 1.0, both containing 1 wt% DMP-30); (**e**) FT-IR spectra of ASB, ECO, and ASB−ECO at various ratios (R = 0.4, 0.6, 0.8, 1.0, and 1.2); (**f**) XPS spectra of ASB-ECO at N1s resolution; (**g**) XPS spectra of ASB−ECO at O1s resolution.

In the experiment, when the molar ratio of ASB to ECO was below 0.4, ASB–ECO could not form a film due to limited crosslinking. The synthesis scheme of ASB–ECO is illustrated in Figure 1a, and the synthesis formulation is provided in Table 1. Taking the synthesis of 1.0-ASB–ECO as an example, 10 g of ECO, 1 wt% of catalyst DMP-30, and 9.323 g of ASB were mixed.

The mixture was then cast into a polytetrafluoroethylene (PTFE) mold and vacuum-cured at 150 °C for 12 h. After cooling to room temperature, a dark brown 1.0-ASB–ECO film was obtained. DBP, a control crosslinker structurally similar to ASB but lacking the Schiff base bond, was added in equimolar amounts to ASB and co-cured with ECO. The control sample 1.0-DBP–ECO was synthesized via an identical protocol to 1.0-ASB–ECO (Figure 1b).

### 2.3. Characterization and Test

#### 2.3.1. FTIR Characterization of ASB and ASB−ECO

Fourier transform infrared (FT−IR) spectra of ASB and ASB−ECO were acquired in ATR mode using an FT-IR spectrometer across the 4000–400 cm^−1^ range. Measurements employed a spectral resolution of 4 cm^−1^ with 32 scans per spectrum.

#### 2.3.2. ^1^H-NMR Characterization of ASB

The ^1^H-NMR spectra of ASB and MDA were acquired on a Bruker Avance III™ HD 500 MHz NMR spectrometer (Bruker, Fällanden, Switzerland) with DMSO-d_6_ as the deuterated solvent.

#### 2.3.3. Differential Scanning Calorimetry (DSC) Characterization of the ASB−ECO Mixture

The curing behavior of ASB−ECO and its curing temperature were assessed using differential scanning calorimetry (DSC, TA−Q20). The ASB–ECO mixture (~5 mg) was loaded into an aluminum crucible and thermally analyzed from −50 to 200 °C at 5 °C/min under controlled heating.

#### 2.3.4. Thermal Stability Test of ASB−ECO

Thermal stability was evaluated using thermogravimetric analysis (TGA, TA Q50). Approximately 5 mg samples in alumina crucibles underwent heating from ambient temperature to 800 °C at 10 °C/min under nitrogen flow.

#### 2.3.5. Dynamic Temperature Analysis of ASB−ECO

The glass transition temperature (Tg) of ASB−ECO at various molar ratios was determined using dynamic mechanical analysis (DMA) (TA-Q800) in single cantilever mode. Samples (35.0 × 12.8 × 3.0 mm) were analyzed at 1 Hz with a heating rate of 5 °C/min from −50 °C to 200 °C.

#### 2.3.6. Stress Relaxation Test of ASB−ECO

Stress relaxation analysis was performed on a TA-Q800 (New Castle, DE, USA) dynamic mechanical analyzer. Samples were heated to target temperatures (80, 90, 100, 110 °C), maintained at 0.01 N static force for 10 min, then subjected to 5% instantaneous strain. The relaxation modulus of the 1.0-ASB−ECO network was monitored at 80, 90, 100, and 110 °C until the relaxation modulus reached equilibrium. The temperature-dependent relaxation time τ* was determined according to the Arrhenius equation:

(1) $$\begin{array}{c} ln\tau^{*}=\frac{E_{a}}{\mathrm{RT}}-lnA \end{array}$$

Here, τ* is the relaxation time, defined as the time for Et/E0 to reach 1/e at a given temperature; Ea is the activation energy of dynamic bond exchange; T is the absolute temperature; R is the molar gas constant; and A is the pre-exponential factor.

#### 2.3.7. Ultraviolet–Visible (UV−Vis) Spectrophotometer Test

UV−vis absorption spectra of ASB, DBP, ASB−ECO, and DBP−ECO were obtained by a PerkinElmer Lambda 750 spectrophotometer (Hopkinton, MA, USA) with a scanning range of 190 to 1100 nm.

#### 2.3.8. Mechanical and Self-Healing Performance Test of ASB−ECO

The mechanical properties of ASB−ECO films before and after recovery were assessed using a TH-8203S universal testing machine (Suzhou, China). Original film samples (45 × 12 × 2 mm) were tested at a stretching rate of 10 mm/min, with each sample tested three times.

Self-healing performance of ASB−ECO was investigated by a TH-8203S electronic universal testing machine. Rectangular specimens (30 mm long, 5 mm wide, and ~0.5–1.0 mm thick) were cut in half through the middle using a security blade. The two sections were then immediately placed in close contact under a certain pressure and exposed to a cold light source of visible light (0.53 W) for 2 h to achieve self-healing. Each sample was tested in parallel three times. Surface temperature during healing was monitored using an infrared thermal imager. The fracture healing rate (ƞ), used to quantify the healing effect, was defined as the percentage of the tensile strength ($\sigma_{t}$) of the healed specimen to the tensile strength ($\sigma_{0}$) of the original specimen:

(2) $$\begin{array}{c} Ƞ=\frac{\sigma_{t}}{\sigma_{0}}\times 100\% \end{array}$$

#### 2.3.9. ASB−ECO Electrical Performance Test

ASB–ECO dielectric characteristics test: The dielectric properties of the ASB−ECO films at normal temperature were tested by a Novocontrol Concept 80 (Ostfildern, Germany) wide temperature and wide frequency impedance analyzer, which operated over a frequency range of 0.1 Hz~1 MHz, test temperature of 25 ± 1 °C, and humidity level below 30%. Each sample was tested three times.

ASB−ECO conductivity characteristics test: An ST2643 ultra-high resistance microcurrent tester (Suzhou, China) equipped with an ST2643-F02 solid three-electrode system featuring a small annular probe was used to assess the electrical insulation performance of the ASB−ECO film. Each sample was tested three times at 25 ± 1 °C.

## 3. Results and Discussion

### 3.1. Structure Characterization of ASB and ASB−ECO

ASB structure characterization: The chemical structure of ASB was confirmed by FT-IR and ^1^H NMR spectroscopy. As shown in Figure 1c, the stretching vibration peak of the aldehyde carbonyl group (C=O) in VAN disappeared at 1660 cm^−1^. Additionally, the four strong N-H stretching vibration peaks of MDA at 3442, 3412, 3334, and 3208 cm^−1^ disappeared in the FT-IR spectrum, while new C=N and C-N stretching vibration peaks appeared at 1608 and 1247 cm^−1^ in ASB, respectively. These observations indicated that VAN underwent a Mannich reaction with MDA, successfully forming a Schiff base bond. Furthermore, the proton peaks for -CHO (10.27 ppm) in VAN and -NH_2_ (4.80 ppm) in both VAN and MDA disappeared in the ^1^H NMR spectra of ASB. Meanwhile, signals corresponding to the protons of the imine, benzene ring, and methoxy groups appeared at 8.43, 7.50–6.87, and 3.95 ppm, respectively (Figure S2). The integral ratios of these peaks aligned with the corresponding number of protons, further confirming the chemical structure of ASB.

Curing and structural characterization of ASB−ECO: To investigate the curing reaction between the hydroxyl groups in ASB and epoxy groups in ECO, as well as the crosslinked network formation, the curing reactivity of ASB and ECO was assessed using DSC in the presence of the DMP-30 catalyst (1 wt%) for transesterification and ring-opening reactions. ASB and dibutyl phthalate (DBP) were used as curing agents for comparison, where the molar ratios of active hydroxyl to epoxy groups (R = ASB/ECO) were set to 0.4, 0.6, 0.8, 1.0, and 1.2. The hydroxyl groups in ASB were found to participate in the curing reaction. Figure 1d presents the DSC curve of the ASB and ECO mixture with 1 wt% DMP-30, which exhibited a significant exothermic curing peak for the ASB–ECO mixture between 50 and 100 °C at various molar ratios. With the increase in ASB content, the peak area gradually increased, which proved that the enthalpy of the reaction increased and the reaction was more sufficient. This indicates that the curing reaction between ASB and ECO occurred at different molar ratios.

The FT-IR spectrum of ASB–ECO (Figure 1e) indicates that the intensity of the epoxy characteristic peak (C-O-C) of ECO at 912 cm^−1^ decreased as the content of ASB increased. This corresponds to the increase in the enthalpy of curing in the DSC test. Concurrently, the characteristic vibration peaks of the imine bond (C=N), ester group (C=O), and methylene group (-CH_2_) in ASB appeared in ASB–ECO at 1608, 1741, and 2855–2924 cm^−1^, respectively. A new hydroxyl (-OH) stretching vibration peak also emerged in ASB–ECO, shifting from 3575 to 3093 cm^−1^, and its peak intensity increased with increasing ASB content. This process indicates that the epoxy groups in ECO etherified with the hydroxyl groups in ASB, forming a permanent ether bond. Figure 1f,g presents the XPS of ASB–ECO, which corresponded to the characteristic peaks of C=N and N-H in ASB at 398.78 and 399.98 eV, respectively, as well as the C=O peak in ECO at 532.18 eV. These results further confirm that the hydroxyl group in ASB underwent a ring-opening reaction with the epoxy group in ECO to form ASB–ECO.

### 3.2. Photothermal Effect and Thermomechanical Properties of ASB−ECO

#### 3.2.1. Photothermal Effect of ASB−ECO

**Figure 2:**
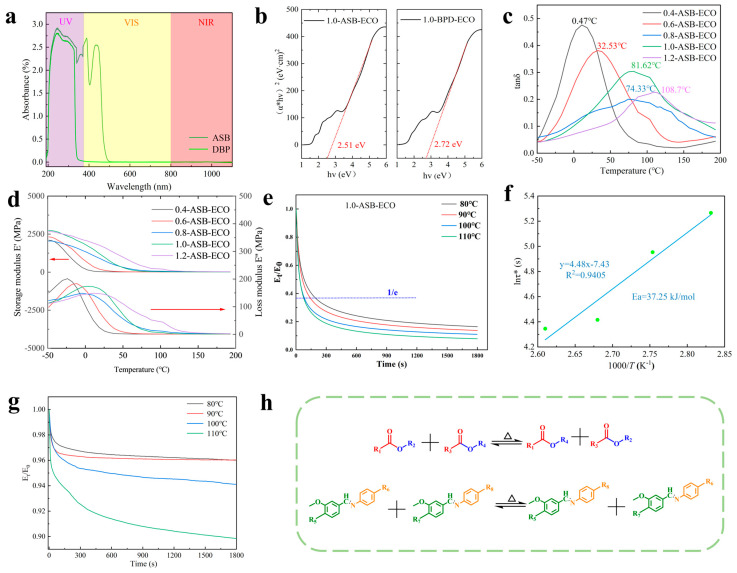
(**a**) UV–vis absorption spectra of ASB and DBP; (**b**) Tauc plots derived from the UV−vis photometer data for 1.0-ASB−ECO and 1.0-DBP−ECO; (**c**) curves of the loss tangent (tan δ) of ASB−ECO at various temperatures; (**d**) curves of the storage modulus (E’) and loss modulus (E”) of ASB−ECO; (**e**) stress relaxation curves for 1.0-ASB−ECO at varying temperatures; (**f**) Arrhenius-type fitted curve based on experimental values of the relaxation time (ln τ*); (**g**) stress relaxation curves for 1.0-DBP−ECO at varying temperatures; (**h**) schematic diagram illustrating dynamic ester bond exchange and dynamic Schiff base bond exchange.

The photophysical properties of ASB and photothermal effects of ASB–ECO in the solid state were subsequently investigated. Previous research has shown that compounds with π–π-conjugated structures exhibit excellent chemical, thermal, and photostable properties [48]. The π delocalization system facilitated π–π stacking, enabling the extension of the absorption spectrum for efficient visible light absorption, while photothermal conversion could be effectively achieved during aggregation. As shown in Figure 2a, ASB in ethanol solution exhibited two strong absorption peaks at 388 and 436 nm. By contrast, the control compound DBP, which had a similar structure but lacked an imine bond, showed no absorption peaks in the visible light range. This indicated that the introduction of the imine structure effectively extended the absorption spectrum into the visible range, allowing ASB to exhibit a favorable response to visible light. According to the energy gap law, a smaller energy gap promoted non-radiative decay, generating heat [49].

To further investigate the photophysical properties of ASB, ASB, and DBP were both thermally cured with ECO. As shown in Figure S6a,b, the incorporation of ASB and DBP effectively extended the absorption spectrum of the polymer to encompass the UV−vis and near-infrared (NIR) regions, resulting in a broad absorption spectrum ranging from 190 to 1100 nm. Notably, within the 400−800 nm range, the 1.0-ASB–ECO composite (2.51 eV), which contained dual dynamic covalent bonds, exhibited a lower bandgap than the 1.0-DBP–ECO composite (2.72 eV) (Figure 2b). According to the energy gap law, a smaller bandgap is typically associated with a faster non-radiative decay rate [50]. These findings suggest that the introduction of imine bonds in ASB enhanced the photothermal conversion efficiency of ASB−ECO and significantly promoted the effective collection of visible light. The photothermal performance of ASB–ECO was quantitatively analyzed by rapidly recording the temperature changes using a Testo thermal infrared imager. As demonstrated in Figure S8a, the 1.0-ASB–ECO film exhibited rapid temperature elevation under visible light irradiation (0.53 W), reaching a thermal equilibrium state at 133 °C within 187 s. Corresponding photothermal performance analysis reveals a remarkable photothermal conversion efficiency (${ƞ}_{PT}$) of 61.42%, as quantified in Figure S8b. In contrast, the temperature of the 1.0-DBP–ECO film in the control group only reached 114 °C (Figure S8c), resulting in a photothermal conversion efficiency (${ƞ}_{PT}$) of 24.93% (Figure S8d). These results indicate that the low energy gap of ASB−ECO significantly enhanced non-radiative decay, as described by the energy gap law, which, in turn, improved the photothermal conversion efficiency of ASB–ECO in the solid state.

#### 3.2.2. Viscoelastic Analysis of ASB−ECO

The dynamic viscoelastic behavior of the ASB–ECO network (R = 0.4, 0.6, 0.8, 1.0, and 1.2) were systematically investigated using dynamic mechanical analysis (DMA). As shown in Figure 2c, each sample exhibited a distinct transition from glass to rubber, with Tg values of 0, 33, 70, 81, and 85 °C. Taking 1.0-ASB–ECO as an example, the storage modulus (E’) was greater than the loss modulus (E”) at room temperature, indicating that elastic deformation dominated over viscous behavior. Furthermore, as Figure 2d illustrates, at temperatures exceeding 120 °C, E’ was comparable to E”, suggesting that viscous deformation predominated. This indicates that elevated temperatures could significantly accelerate the bond exchange reactions of dynamic covalent bonds, facilitating network topology reorganization and enhancing self-healing performance.

#### 3.2.3. Stress Relaxation Analysis

To evaluate the bond exchange reaction capabilities of dynamic covalent bonds, specifically ester bonds and Schiff bases in ASB−ECO at elevated temperatures, we measured the stress relaxation modulus and temperature dependence of the 1.0−ASB−ECO film and 1.0-DBP–ECO control sample, which lacked imine bonds, under 5% strain. The relaxation time (τ*) is defined as the duration required for the normalized relaxation modulus (E_t_/E_0_) to decline to 1/e, which is based on the assumption that terminal relaxation could be approximated by the Maxwell model. Figure 2g illustrates the variation in normalized relaxation modulus (E_t_/E_0_) for the control group (1.0-DBP−ECO) across a temperature range of −50 to 200 °C. The findings indicated that 1.0-DBP–ECO did not achieve complete stress relaxation at different temperatures during the same heat treatment duration, suggesting that the bond exchange reaction was constrained at elevated temperatures, thereby exhibiting residual viscoelastic behavior. Figure 2e presents the variation in normalized relaxation modulus (Et/E0) for the 1.0-ASB–ECO film as a function of relaxation time (τ*), defined as the duration required for Et/E0 to decrease to 1/e. As the temperature increased, τ* gradually decreased in correlation with the reduction in normalized relaxation modulus. Specifically, the relaxation times (τ*) for the 1.0-ASB–ECO film were measured as 194 s at 80 °C, 142 s at 90 °C, 83 s at 100 °C, and 77 s at 110 °C. A strong linear relationship was observed between ln(τ*) and 1000/T. The topology freezing temperature (Tv), defined as the temperature at which the extrapolated viscosity reached approximately 10^12^ Pa∙s [33,51,52], was calculated as 123.13 °C for 1.0-ASB–ECO. Additionally, the bond exchange activation energy (Ea) at Tv was 37.2541 kJ/mol (Figure 2f). This behavior could be attributed to the fact that, upon reaching Tv, the ASB–ECO segments acquired sufficient free volume to facilitate both local and long-range movement. Consequently, the dual dynamic covalent bonds within ASB–ECO were activated by heat, enabling dynamic reversible exchange reactions. The presence of these dual dynamic covalent bonds resulted in a more rapid bond exchange response and enhanced temperature response. These findings suggested that ASB−ECO exhibited significant viscoelastic behavior and remarkable self-healing capabilities at elevated temperatures.

### 3.3. ASB−ECO Mechanical Properties and Self-Healing Properties

**Figure 3:**
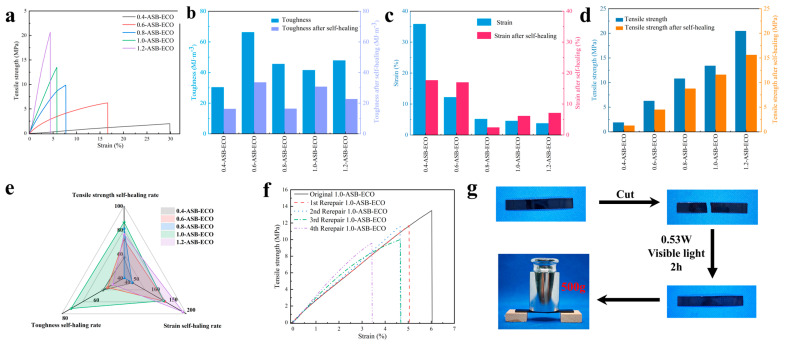
(**a**) Stress−strain curves of ASB−ECO; histogram of ASB−ECO toughness (**b**), strain (**c**), and tensile strength (**d**) before and after self-healing; (**e**) radar charts of the repair toughness, strain, and tensile strength self-healing rates of ASB−ECO; (**f**) stress−strain curves of 1.0-ASB−ECO after four healing cycles; (**g**) picture of the 1.0-ASB−ECO film supporting heavy objects after fracture and self-healing, following exposure to visible light (0.53 W) for 2 h.

The tensile performances of the ASB−ECO films with varying ASB contents were evaluated, as illustrated in Figure 3a. When the ASB−ECO ratio was increased from 0.4 to 1.0, the tensile strength of the ASB–ECO films increased from 1.976 to 20.67 MPa, while the toughness improved from 30.33 to 47.83 MJ/m^3^. The incorporation of rigid ASB groups within the polymer matrix contributed to these enhancements in mechanical properties. Conversely, the breaking elongation of the ASB–ECO films decreased from 29.88 to 4.39%. This reduction was a result of the increased crosslinking density of the polymer chains at higher ASB contents, which restricted the mobility of the macromolecular chains. This phenomenon was further supported by the observed increase in the Tg of the ASB–ECO films. Additionally, as shown in Figure 2d, the storage modulus (E′) of the ASB–ECO films increased with ASB content at room temperature, further confirming the enhancement in mechanical properties.

Considering the excellent photothermal conversion capabilities and dynamic reversibility of the ASB−ECO films, we further investigated their fracture self-healing properties based on photothermal effects. The tensile strength, strain, and toughness healing rates of the healed ASB−ECO films are illustrated in Figure 3b–e. Radiation treatment was applied to the reconnected ASB−ECO films for 2 h after they were cut, using a cold light source (380–800 nm, 0.53 W). The results indicate that both the tensile strength and toughness of the samples increased with increasing ASB content. However, the healing rate initially increased before subsequently decreasing. The strain healing rate of ASB−ECO also demonstrated an initial increase, followed by a decrease, and then a subsequent rise. Notably, the tensile strength and toughness of the 1.0-ASB−ECO reached maximum values of 86.52 and 74.04%, respectively. When the ASB content was appropriately increased, the strain healing rate of the dynamic crosslinked network in ASB−ECO gradually increased from 49.35 to 139.53%. However, with further increases in ASB content, the higher degree of crosslinking among the polymer chains restricted the movement of macromolecular chains, leading to a decrease in the strain healing rate from 139.53 to 47.59%. With elevated ASB content, the concentration of dynamic covalent bonds also increased, and when the molar ratio exceeded 1.0, the strain healing rate of ASB−ECO became significant, exceeding 100%. Furthermore, as shown in Figure 3f, the 1.0-ASB−ECO film retained 71% of its original mechanical properties after four fracture-healing cycles.

Due to the dynamic characteristics of ASB−ECO, these films exhibited impressive welding performance. A rectangular 1.0-ASB−ECO film with a thickness of 0.5 mm was completely fractured; however, the reconditioned film could support a 500 g weight, providing visual evidence of the self-healing properties of ASB–ECO (Figure 3g). The self-healing properties of ASB−ECO effectively extended its life cycle, enhancing its sustainability.

### 3.4. Electrical and Thermal Conductivity of ASB−ECO

#### 3.4.1. Dielectric Characteristics Analysis

**Figure 4:**
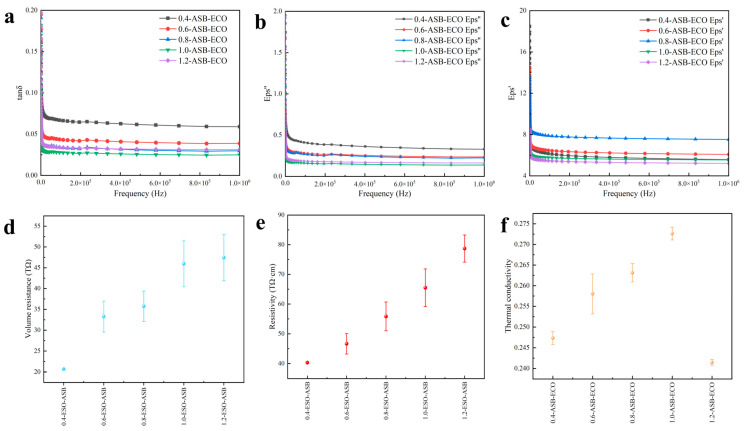
Frequency-dependent curves of the real part (**a**), imaginary part (**b**), and dielectric loss (**c**) of the complex permittivity of ASB–ECO with different contents of ASB; volume resistance (**d**), resistivity and conductivity (**e**) and thermal conductivity (**f**) changes in the ASB–ECO composites with different ASB contents.

With electronic power devices advancing into the high-frequency domain, the transmission speed and loss during signal transmission can be positively correlated with the dielectric constant, making it essential for epoxy molding materials to exhibit a low dielectric constant and low dielectric loss in the high-frequency region. Figure 4a,b illustrates the frequency-dependent curves of the real and imaginary components of the complex permittivity of ASB−ECO at various molar ratios at 25 °C. ASB–ECO exhibited a low dielectric constant across different ASB contents. Additionally, as the molar content of ASB increased, the dielectric constant of ASB−ECO initially decreased and then increased. The complex dielectric constant of the ASB−ECO composites reached its minimum when the molar ratio of ASB to ECO was 1.0. For example, at 10^6^ Hz, the real and imaginary dielectric constants of the ASB−ECO composites declined from maximum values of 7.53 and 0.33, to 5.21 and 0.14, respectively. Figure 4c presents the dielectric loss curve of the ASB−ECO composites, which aligned with the dielectric constant results. When the molar ratio of ASB to ECO was 1.0, the dielectric loss of the ASB−ECO composites was minimized, with a dielectric loss tangent (tan δ) of 0.0249 at 10^6^ Hz. At low frequencies, all dipole groups in the epoxy molecular chain could align themselves. However, as the frequency of the AC voltage increased, the dipole reorientation failed to maintain pace with the changing direction of the electric field, resulting in incomplete stabilization of polarization and a rapid decrease in the dielectric constant of ASB−ECO across different compositions.

#### 3.4.2. Conductivity Characteristics Analysis

Conductivity is a physical quantity that reflects the conductivity of dielectric materials, where the lower the electrical conductivity, the lower the likelihood of electrical conduction, and the better the insulating properties of the material. Figure 4d,e presents the DC conductivity and volume resistance of the ASB−ECO composites at different molar ratios at 30 °C and 100 V. The conductivity of the ASB−ECO composites was 0.01274 pS/cm with increasing ASB content, and the maximum resistance was 78.7 TΩ·cm. The resistivity of ASB–ECO was 40.31–78.7 times that of some ordinary epoxy resins (0.01–1 TΩ·cm). The results show that the filling of ASB could promote an increase in the resistance of the epoxy resin. The volume resistance results further demonstrate that the increase in ASB content further improved the insulation properties of the ASB−ECO composites, and the volume resistance increased from 20.66 to 47.43 TΩ. This could be attributed to the chemical reaction between ASB and ECO, where the resulting dense crosslinking limited the migration of carriers.

## 4. Conclusions

In this study, we propose a method for preparing castor oil-based epoxy vitrimers (ASB−ECO) that are suitable for electronics, featuring high mechanical properties, photothermal conversion, and self-healing. The dual dynamic covalent adaptive network structure of 1.0-ASB−ECO contributes to a high tensile strength of 20.67 MPa and facilitates topological network rearrangement, with a relaxation time (τ*) of 77 s at 110 °C, a topological freezing transition temperature (Tv) of 123.13 °C, and an activation energy (Ea) of 37.25 kJ/mol. The material can maintain 71% of its original mechanical properties after four fracture-healing cycles. The incorporation of an aromatic imine structure crosslinker imparts photothermal repair capabilities, demonstrating a photothermal conversion efficiency (${ƞ}_{PT}$) of 61.42%. Additionally, ASB−ECO exhibits a low dielectric constant and dielectric loss, along with high electrical insulating properties. This work provides an innovative approach for preparing multifunctional bio-based epoxy resin vitrimers that help address the need for sustainable materials in advanced electronics, especially helping solve the challenges related to longevity and repairability.

## Figures and Tables

**Table 1:** Compositions for preparing ASB–ECO and DBP–ASB.

Sample Codes	ASB (g)	DBP (g)	ECO (g)
0.4-ASB–ECO	3.72	0	10
0.6-ASB–ECO	5.29	0	10
0.8-ASB–ECO	7.65	0	10
1.0-ASB–ECO	9.32	0	10
1.2-ASB–ECO	11.18	0	10
1.0-DBP–ECO	0	7.44	10

## Data Availability

The original contributions presented in this study are included in the article and Supplementary Materials. Further inquiries can be directed to the corresponding author.

## Supplementary Materials

The following supporting information can be downloaded at: https://www.mdpi.com/article/10.3390/polym17070897/s1, Figure S1: Schematic diagram of ASB synthesis; Figure S2: ^1^H NMR spectrum of VAN, MDA, and ASB; 400 MHz, DMSO-d_6_; Table S1: Compositions for preparing ASB–ECO and DBP–ASB; Figure S3: (a) XPS broad-spectrum of ASB–ECO; (b) resolution XPS spectra of O1s in ASB–ECO; (c) N1s resolution XPS spectra; (d) high-resolution XPS spectra of C1s; Figure S4: (a) TGA curves of ASB and ECO; (b) DTG curves of ASB versus ECO; (c) TGA curves of ASB–ECO at each mole; (d) DTG curves of ASB–ECO at each mole; Figure S5: The Arrhenius equation after fitting according to the relaxation time (τ*); Figure S6: UV–vis absorption spectra of 1.0-ASB–ECO (a) and 1.0-DBP–ECO (b) composites; Tauc plot curve calculated from UV–vis photometer data of 1.0-ASB–ECO (c) and 1.0-DBP–ECO (d) composites; Figure S7: Variation curves of specific heat capacity Cp of samples at different temperatures; Figure S8: (a) Heating and cooling curves of 1.0-ASB–ECO films under 400–800 nm xenon lamp irradiation; (b) linear fitting relationships between time △t and -lnθ obtained from the cooling range of 1.0-ASB–ECO films; (c) heating and cooling curves of 1.0-DBP–ECO films under 400–800 nm scientific xenon lamp irradiation; (d) the linear fitting relationship between time △t and -lnθ obtained from the cooling interval of 1.0-DBP–ECO films; Figure S9: The 1.0-ASB–ECO spline before and after 1 week in water, ethanol, and THF; Figure S10: Variation in thermal conductivity of ASB–ECO at different moles; Figure S11: Comparison of tensile strength, Tg obtained from DMA, and activation energy Ea for 1.0-ASB–ECO and previously reported vegetable oil-based vitrimers; Table S2: Properties of the reported vegetable oil-based vitrimers. References [53,54,55,56,57] are cited in the supplementary materials.
