# Supplementary material for: Castor Oil-Based Epoxy Vitrimer Based on Dual Dynamic Network with Intrinsic Photothermal Self-Healing Capability
Source: Polymers (Basel). 2025 Mar 27;17(7):897. doi: 10.3390/polym17070897 (PMC11991231; doi:10.3390/polym17070897)
Supplement: Supplementary file 1 [file polymers-17-00897-s001.zip › polymers-3525375-supplementary.pdf]

Supporting Information

Castor Oil-Based Epoxy Vitrimer Based on Dual Dynamic Network with Intrinsic Photothermal Self-Healing Capability

Yingqing Shao [1,†], Haoxin Zhu [1,†], Kang Chen [1], Tianyi Jin [1,2], Zhiwen Wang [1,2], Zhixin Luo [1,2], Jinhui Wang [1,2], Haoyuan Sun [1,2], Shuangying Wei [1,2,3,*] and Zhenhua Gao [1,2,3,*]

[1] College of Material Science and Engineering, Northeast Forestry University, Harbin 150040, China; 2022111431@nefu.edu.cn (Y.S.); haoxin.zhu24@outlook.com (H.Z.); kangchen@hrbeu.edu.cn (K.C.); 15033797211@163.com (T.J.); 19163346882@163.com (Z.W.); 18755668280@163.com (Z.L.); 19945941145@163.com (J.W.); gibrilasu5@gmail.com (H.S.)

[2] Engineering Research Center of Advanced Wooden Materials, Ministry of Education, Northeast Forestry University, Harbin 150040, China

[3] Key Laboratory of Bio-Based Material Science & Technology, Ministry of Education, Northeast Forestry University, Harbin 150040, China

* Correspondence: dephnewsy@nefu.edu.cn (S.W.); gaozh@nefu.edu.cn (Z.G.)

† These authors contributed equally to this work.

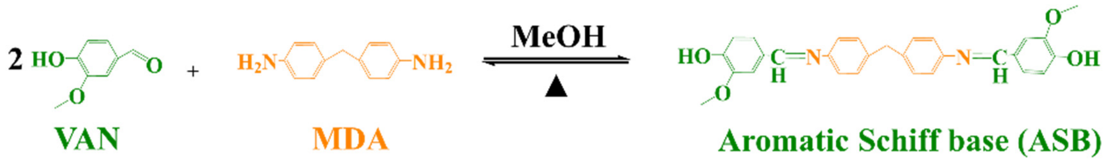

Figure S1. Schematic diagram of ASB synthesis.

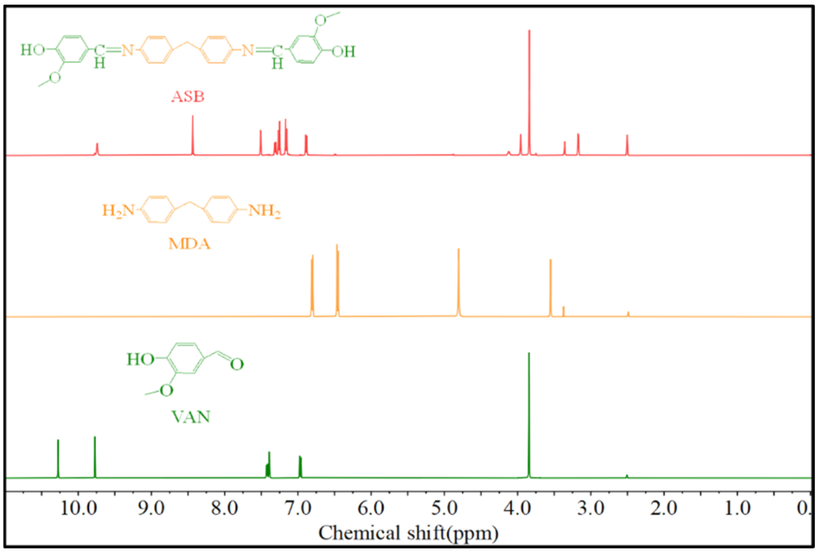

Figure S2. ^{1}H NMR spectrum of VAN, MDA, and ASB; 400 MHz, DMSO-d_6.

Table S1. Compositions for preparing ASB–ECO and DBP–ASB.

Sample Codes	ASB (g)	DBP (g)	ECO (g)
0.4-ASB–ECO	3.72	0	10
0.6-ASB–ECO	5.29	0	10
0.8-ASB–ECO	7.65	0	10
1.0-ASB–ECO	9.32	0	10
1.2-ASB–ECO	11.18	0	10
1.0-DBP–ECO	0	7.44	10

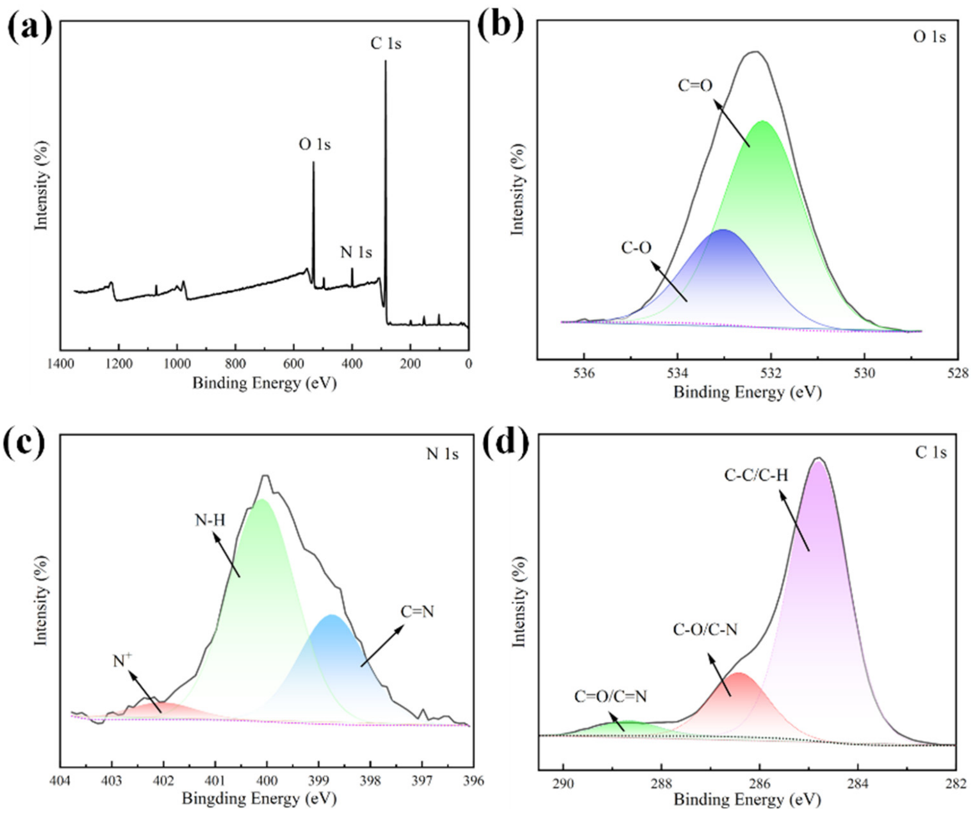

Figure S3. (a) XPS broad-spectrum of ASB–ECO; (b) resolution XPS spectra of O1s in ASB–ECO; (c) N1s resolution XPS spectra; (d) high-resolution XPS spectra of C1s.

TGA Characterization

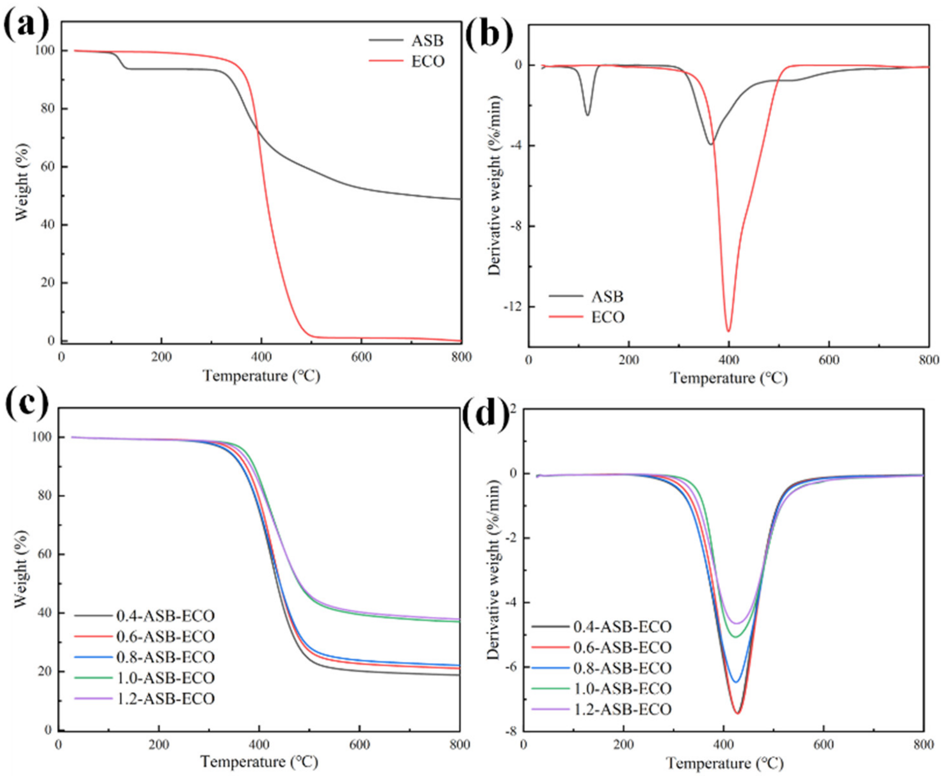

Figure S4. (a) TGA curves of ASB and ECO; (b) DTG curves of ASB versus ECO; (c) TGA curves of ASB–ECO at each mole; (d) DTG curves of ASB–ECO at each mole.

In order to ensure that ASB–ECO does not degrade the polymer due to high temperature during the subsequent self-healing process, the thermal stability of ASB, ECO, and ASB–ECO–TBD mixtures was investigated using TG.

As can be seen from Figures S4a,b, the mass fraction (TGA) curve of the ASB specimen shows two decomposition peaks at 80 °C and 290 °C, corresponding to the raw material and ASB decomposition temperature, respectively. The mass fraction of the ECO was stable until 242 °C, and when the temperature exceeded 242 °C, the mass fraction of the ECO specimen began to decrease significantly, while the decomposition temperature of the ASB–ECO was 300 °C (Figures S4c,d). The results show that the introduction of ASB helps to reduce the mass loss of epoxy resins. In addition, the mass differential (DTG) curve also shows that the loss of mass fraction of ASB–ECO composites gradually decreased with the increase in ASB content.

Stress relaxation characterization

Determine the freezing transition temperature (T_v) of the topological network and the activation energy (E_a) for bond exchange.

The 1.0-ASB–ECO film was subjected to a stress relaxation test. The equation is linearly fitted from the Arrhenius equation:

$$y=4.48035x-7.43608\left(R^2=0.9405\right) \quad \text{(S1)}$$

y stands for $\ln\tau^*$, and x stands for $\frac{1000}{T_v}$. To calculate T_v using the Maxwell relationship, T_v is defined as the temperature at which the viscosity of the material is 10^{12} Pa·s and the material relaxes to $\frac{1}{e}$ of its initial modulus. The relaxation modulus, G, is estimated using the energy storage modulus (E'). According to the temperature–modulus curve, the

average E' modulus of the sample stabilizes at 4.739 MPa after being heated to 51.09 °C (figure 2d). The Poisson's ratio μ in the equation is assumed to be 0.5.

According to Equations (S2) and (S3) as follows:

$$\eta = G \times \tau^* \quad \text{(S2)}$$

$$G = \frac{E'}{2(1+\mu)} \quad \text{(S3)}$$

Obtain the value of G and τ^*

$$G = \frac{4.739}{2(1+0.5)} = 1.5797 \times 10^6 \text{ (Pa)}$$

$$\tau^* = \frac{\eta}{G} = \frac{10^{12}}{1.5797 \times 10^6} = 6.33031 \times 10^5 \text{(s)}$$

Put the value in Equation (S1)

$$\ln\tau^* = \ln(6.33031 \times 10^5) = 13.35827 \text{(min)}$$

$$13.35827 = 22.96x - 44.58,\quad x = 2.52344$$

Obtain the value of T_v

$$T_V = \frac{1000}{x} - 273.15 = 123.1344°C$$

E_a is obtained by linearly fitting the slope (K) of the equation

$$K = \frac{E_a}{R} = 4.48035,\quad E_a = 37.2541 \text{ kJ/mol}$$

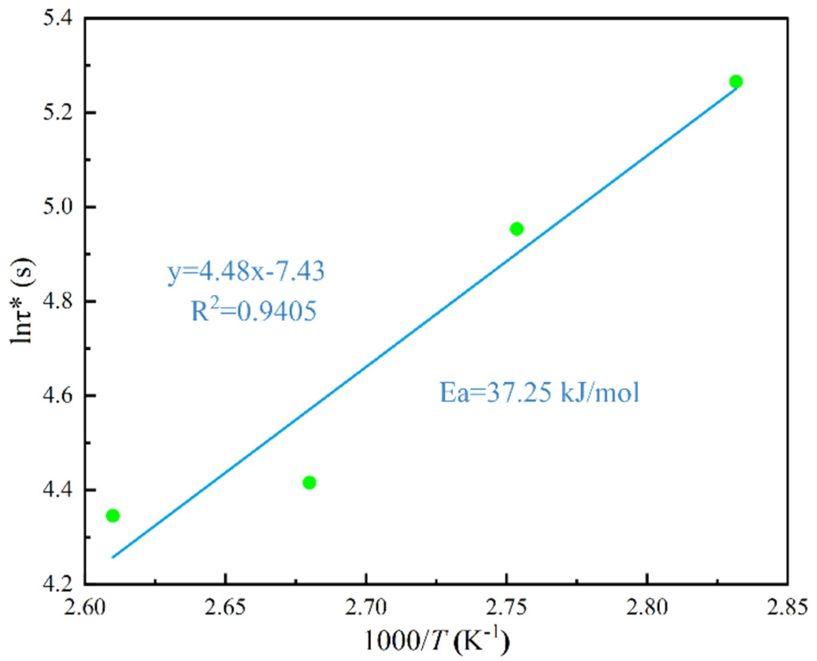

Figure S5. The Arrhenius equation after fitting according to the relaxation time (τ*).

UV–vis absorption spectra of ASB–ECO and Tauc plot method to calculate the bandgap width value (Eg)

According to Figure S6a–b, the ultraviolet–visible photometer test data are $(\alpha h\nu)^{\frac{1}{n}}$ and hν, respectively, mainly using the Tauc plot equation:

$$(\alpha h\nu)^{\frac{1}{n}} = A(h\nu - E_g) \quad (S4)$$

Among them, α is the absorbance index, and because the absorbance is proportional to the absorbance coefficient, the absorbance is used instead of the absorbance coefficient; h is Planck's constant, ν is the frequency, A is the constant, and Eg is the semiconductor bandgap width. The exponent n is directly related to the type of semiconductor, and the direct bandgap semiconductor n = 1/2; indirect bandgap semiconductors n = 2.

Because

$$h\nu = \frac{hc}{\lambda} \quad (S5)$$

where h = 6.63 × 10^{-34} J·s, c is the speed of light 3 × 10^{-8} m·s^{-1}, λ is the wavelength of light. It can be obtained:

$$h\nu = \frac{1240}{\lambda}$$

hν is plotted with $(\alpha h\nu)^{\frac{1}{n}}$. The tangent extension of the straight line is made to the abscissa axis (y = 0), and the intersection point is the bandgap width value Eg. As shown in Figure S6c,, the Eg of 1.0-ASB–ECO and 1.0-DBP–ECO are 2.51 eV and 2.72 eV.

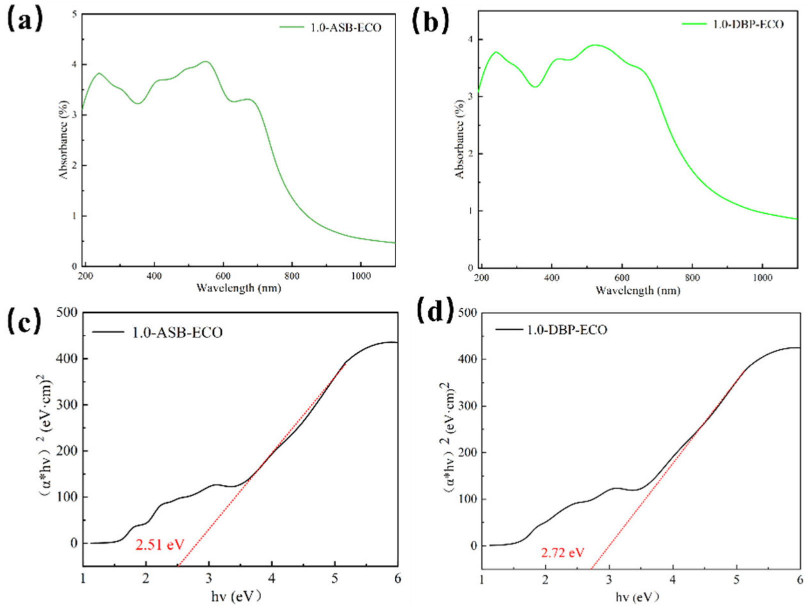

Figure S6. UV–vis absorption spectra of 1.0-ASB–ECO (a) and 1.0-DBP–ECO (b) composites; Tauc plot curve calculated from UV–vis photometer data of 1.0-ASB–ECO (c) and 1.0-DBP–ECO (d) composites.

DSC sapphire specific heat capacity test

Using the TA DSC250, the specific heat capacity of the sample is calculated by measuring the heat flux of the sapphire standard and the sample, and combining the proportional relationship between the standard sample and the sample. Specific method of measuring specific heat capacity: sapphire 26.5 mg; mode: modulation; trial: conventional MDSC; modulation amplitude: ±1.0 °C; modulation period: 120 s. The average heating rate was 3 °C/min. Therefore, the temperature range selected for the calibration is between -50 °C and 100 °C.

$$C_P = \frac{C'_P m' Q}{m Q'} \quad \text{(S6)}$$

Where Cp is the specific heat capacity of the sample, J/(g·k); Cp′ is the specific heat capacity of the sapphire standard, J/(g·K); m is the mass of the sample, 8.94mg; m′ is the mass of the sapphire standard, 23.6mg; Q is the heat flux of the sample, W; Q′ is the heat flux of the sapphire standard, W.

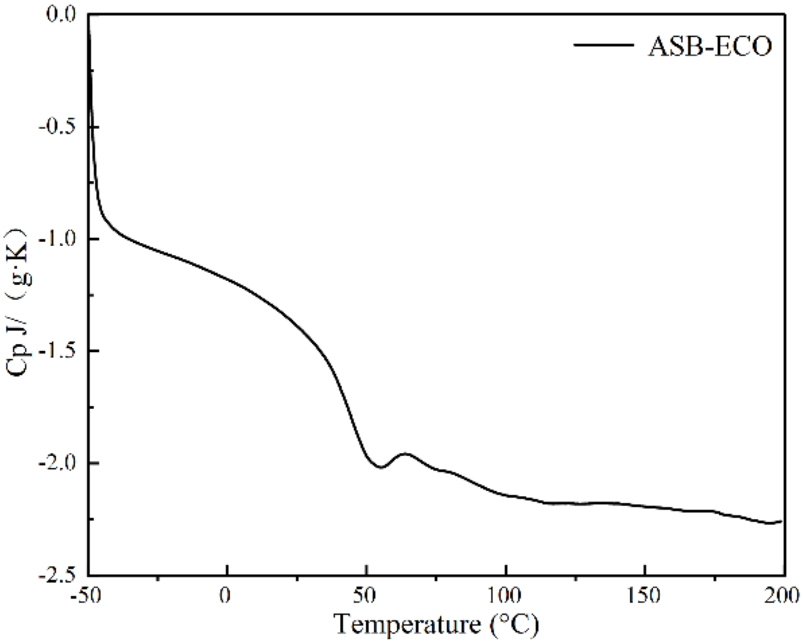

Figure S7. Variation curves of specific heat capacity Cp of samples at different temperatures.

Photothermal conversion efficiency of ASB–ECO films

The photothermal properties of ASB–ECO and DBP–ECO films under 400–800nm xenon lamps with a power of 0.53W were investigated, and the surface temperature was recorded by a thermal infrared imager.

The photothermal conversion efficiency of the 1.0-ASB–ECO film was explored, and the photothermal conversion efficiency η_{PT} at fixed light power (0.53W) was calculated according to Equation (S7):

$$\eta_{PT} = \frac{hA\Delta T_{max}}{I} \times 100\% \quad \text{(S7)}$$

where h is the heat transfer coefficient, A is the surface area of the system, ΔT_{max} is the temperature difference between the maximum steady-state temperature and the ambient temperature, and I is the light power.

The height of the scientific xenon lamp (400–800 nm) from the surface of the membrane is 21.08 mm and the diameter is 1.487 cm, while the diameter of the 1.0-ASB–ECO

membrane is 0.814 cm, so the power irradiated at the surface of the 1.0-ASB–ECO membrane is 0.2901 W according to the area ratio; hA can be determined from the linear-fit data for the cooling period t–lnθ. At the same time, the value of hA is derived according to Equation (S8):

$$t = \frac{\sum_i m_i C_{p,i}}{h_A} \ln\theta \quad \text{(S8)}$$

θ is the ratio of △T to $△T_{max}$, and C is the specific heat capacity at Tv obtained by DSC test of sample (8.59mg) by sapphire method.

The specific heat (Cp) of the 1.0-ASB–ESO film at Tv (123.13°C) in Figure S7 is 2.1794 J/(g·k), hA is calculated to be 4.5985×10^{-4} J/(K·s), and the photothermal conversion rate η_{PT} is 61.42·%.

Similarly, since the 1.0-DBP–ESO film does not have Tv, only Tg, the specific heat (Cp) of the 1.0-DBP–ESO film at Tg is 1.4954 J/(g·k), hA is calculated to be 1.9551×10^{-4} J/(K·s), and the photothermal conversion rate η_{PT} is 24.93%.

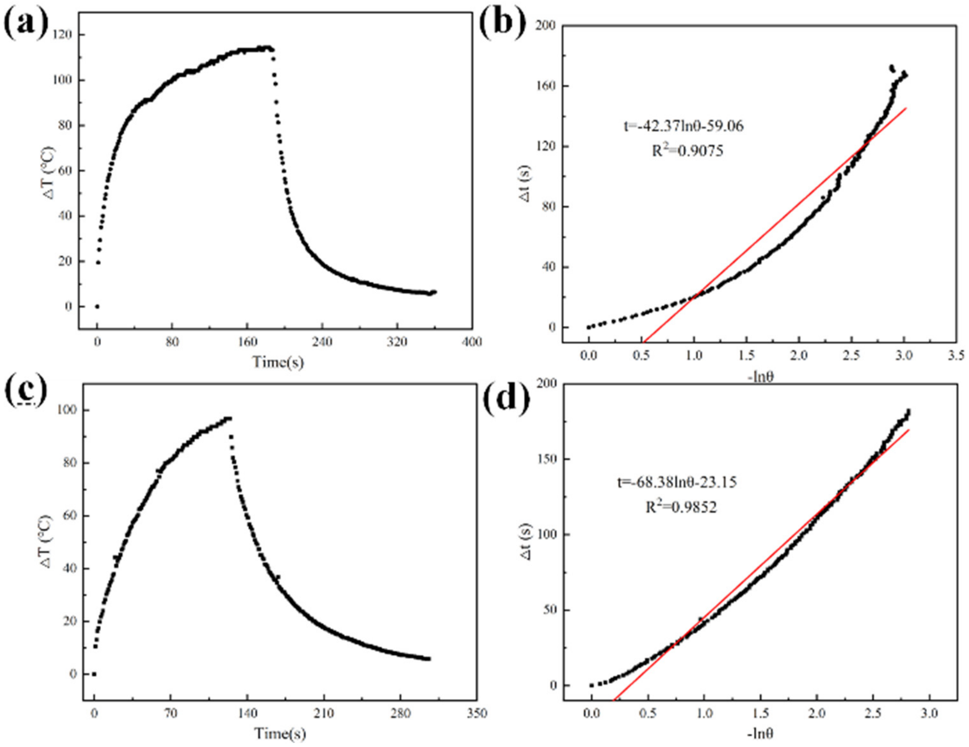

Figure S8. (a) Heating and cooling curves of 1.0-ASB–ECO films under 400–800 nm xenon lamp irradiation; (b) linear fitting relationships between time △t and -lnθ obtained from the cooling range of 1.0-ASB–ECO films; (c) heating and cooling curves of 1.0-DBP–ECO films under 400–800 nm scientific xenon lamp irradiation; (d) the linear fitting relationship between time △t and -lnθ obtained from the cooling interval of 1.0-DBP–ECO films.

Solvent resistance of ASB–ECO films

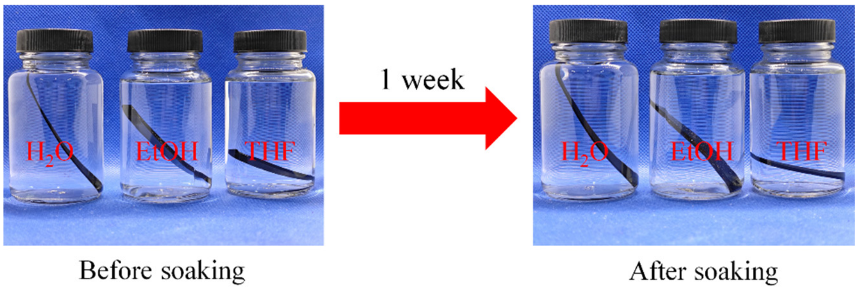

Figure S9. The 1.0-ASB–ECO spline before and after 1 week in water, ethanol, and THF.

Thermal conductivity characterization of 1.0-ASB–ECO

For electronic materials, the improvement in thermal conductivity (TC) is one of the important indicators that insulating materials can be used for power electronic device. Thermal conductivity is determined with reference to ISO22007-2. The instrument TPS 2500 S is manufactured by Hot Disk Company in Sweden. The thermal conductivity of the thin-film specimen was measured using the thin-film measurement module. The test probe in this article uses the 5501 6.403 mm Kapton Sensor, which uses a radial detection depth of 15 mm.

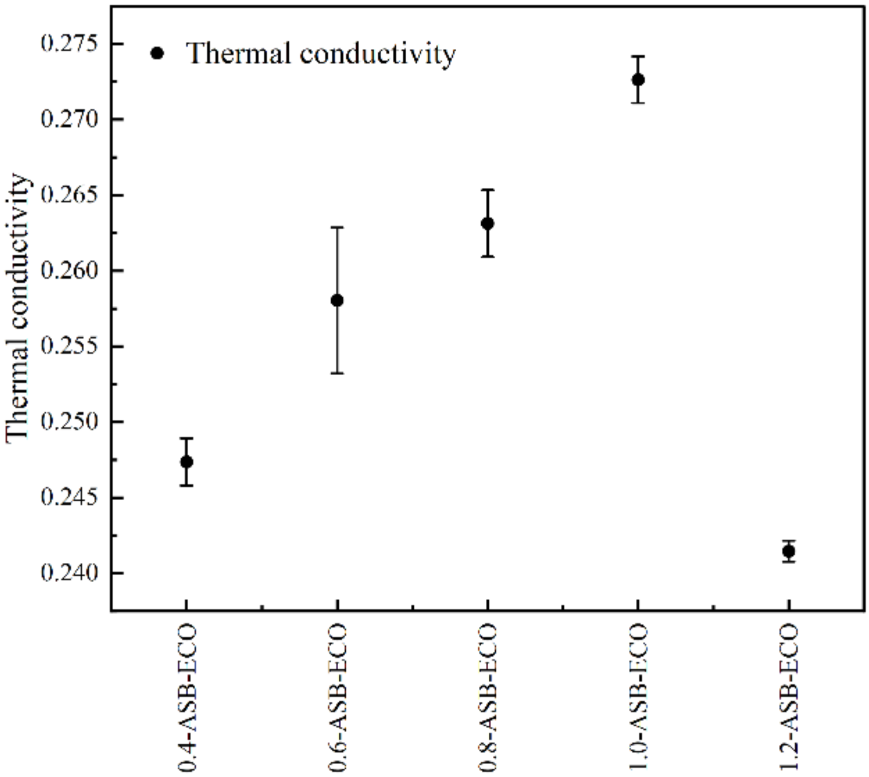

Figure S10. Variation in thermal conductivity of ASB–ECO at different moles.

For electronic materials, the improvement in thermal conductivity (TC) is one of the important indicators that insulating materials can be used for power electronic device packaging. As a traditional amorphous polymer, epoxy resin is intricately intertwined with the internal molecular chains, which makes the free travel of phonons short, the heat cannot be quickly exported, and the thermal conductivity is low. The thermal conductivity and lifting rate of ASB–ECO composites are shown in Figure S10, and the thermal

conductivity of the sample increases first and then decreases with the increase in ASB content. This is due to the fact that with the increase in the ASB content of the conjugated structure, the isotropic orientation and high cross-linking density in the ASB–ECO film further increase, which further promotes the heat transfer within the phononic polymer, but with an R of 1.2, the high-filler ASB will form cavities and bring more impurities between the ASB–ECO composites, resulting in phonon scattering and a decrease in thermal conductivity.

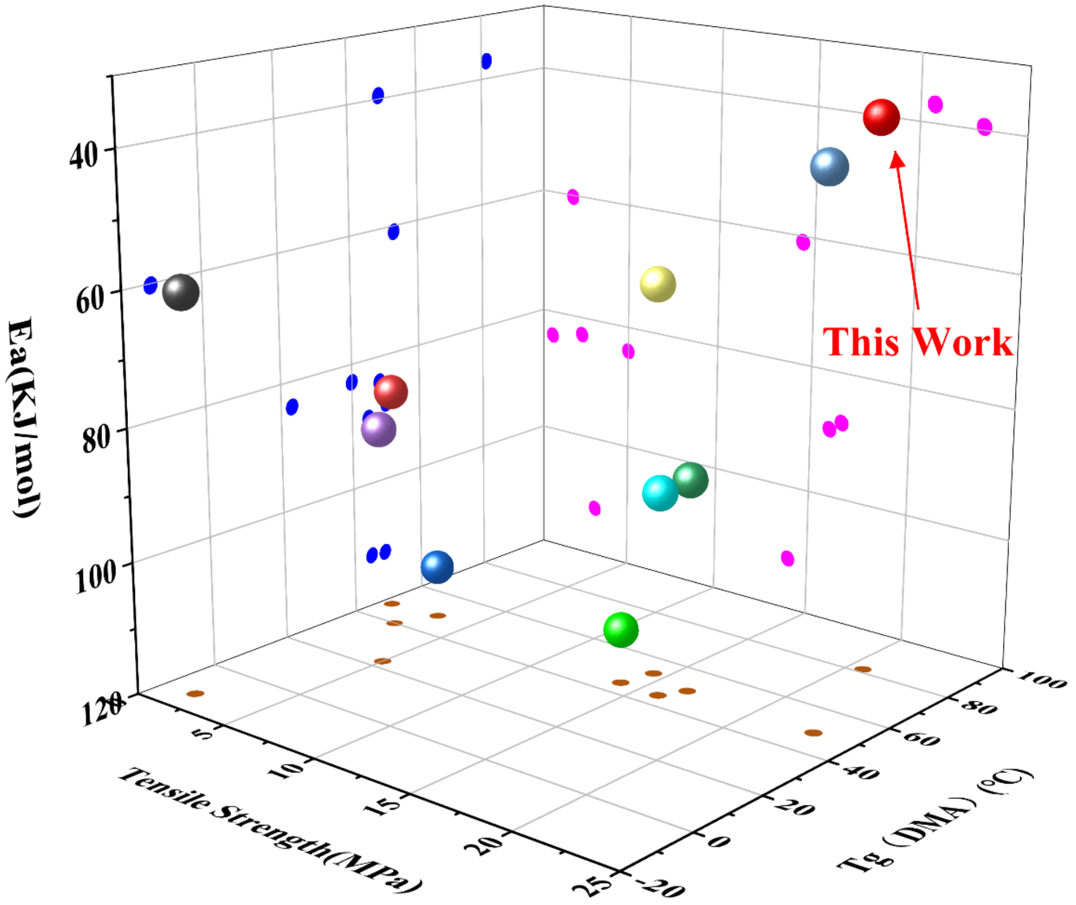

Figure S11. Comparison of tensile strength, Tg obtained from DMA, and activation energy Ea for 1.0-ASB–ECO and previously reported vegetable oil-based vitrimers.

Table S2. Properties of the reported vegetable oil-based vitrimers.

Reference	Tensile Strength (MPa)	Tg Obtained From DMA (°C)	Ea (KJ/mol)
Soybean oil and divanillic [1]	14	45°C	111.8
Soybean oil and camphoric [2]	0.56	48	84
Soybean oil and glycyrrhizic [3]	3.1	49	112
Epoxy soybean oil and fumaropimaric acid [4]	16.62	50	87.88
Epoxidized soybean oil and sugar alcohols [5]	5	23	83.95
Epoxidized linseed oil and boronic ester [6]	23	49	39.5
Epoxidized soybean oil with doubly esterified starch [7]	1.75	-12.81	60.27
Epoxidized methacrylated castor oil and itaconic acid [8]	14.39	52.95	60.94
Epoxidized soybean oil and 4, 4′-dithiodiphenylamine [9]	2.3	40	82.85
Epoxidized methacrylated castor and itaconic acid and 4,4′-dithiodiphenylamine [10]	16	44.6	89.29
This work	20.67	81.62	37.25

References

1. Zhang, Y.; Yukiko, E.; Tadahisa, I. Bio-based vitrimers from divanillic acid and epoxidized soybean oil. *RSC Sustain.* **2023**, *1*, 543–553. https://doi.org/10.1039/D2SU00140C.
2. Zhang, W.; Wu, J.; Gao, L.; Zhang, B.; Jiang, J.; Hu, J. Recyclable, reprocessable, self-adhered and repairable carbon fiber reinforced polymers using full biobased matrices from camphoric acid and epoxidized soybean oil. *Green Chem.* **2021**, *23*, 2763–2772. https://doi.org/10.1039/D1GC00648G.
3. Wu, J.; Yu, X.; Zhang, H.; Guo, J.; Hu, J.; Li, M.-H. Fully Biobased Vitrimers from Glycyrrhizic Acid and Soybean Oil for Self-Healing, Shape Memory, Weldable, and Recyclable Materials. *ACS Sustain. Chem. Eng.* **2020**, *8*, 6479–6487. https://doi.org/10.1021/acssuschemeng.0c01047.
4. Yang, X.; Guo, L.; Xu, X.; Shang, S.; Liu, H. A fully bio-based epoxy vitrimer: Self-healing, triple-shape memory and reprocessing triggered by dynamic covalent bond exchange. *Mater. Des.* **2020**, *186*, 108248. https://doi.org/10.1016/j.matdes.2019.108248.
5. Li, J.; Ju, B.; Zhang, S. Catalyst-free, sustainable epoxy vitrimers from epoxidized soybean oil and natural sugar alcohols. *Ind. Crops Prod.* **2023**, *205*, 117466. https://doi.org/10.1016/j.indcrop.2023.117466.
6. Sangaletti, D.; Ceseracciu, L.; Marini, L.; Athanassiou, A.; Zych, A. Biobased boronic ester vitrimer resin from epoxidized linseed oil for recyclable carbon fiber composites. *Resour. Conserv. Recycl.* **2023**, *198*, 107205. https://doi.org/10.1016/j.resconrec.2023.107205.
7. Li, C.; Ju, B.; Zhang, S. Fully bio-based hydroxy ester vitrimer synthesized by crosslinking epoxidized soybean oil with doubly esterified starch. *Carbohydr. Polym.* **2023**, *302*, 120442. https://doi.org/10.1016/j.carbpol.2022.120442.
8. Zhang, Y.; Zhai, M.; Shi, L.; Lei, Q.; Zhang, S.; Zhang, L.; Lyu, B.; Zhao, S.; Ma, J.; Thakur, V.K. Sustainable castor oil-based vitrimers: Towards new materials with reprocessability, self-healing, degradable and UV-blocking characteristics. *Ind. Crops Prod.* **2023**, *193*, 116210. https://doi.org/10.1016/j.indcrop.2022.116210.
9. Liu, Y.-Y.; He, J.; Li, Y.-D.; Zhao, X.-L.; Zeng, J.-B. Biobased, reprocessable and weldable epoxy vitrimers from epoxidized soybean oil. *Ind. Crops Prod.* **2020**, *153*, 112576. https://doi.org/10.1016/j.indcrop.2020.112576.
10. Zhang, Y.; Zhang, S.; Zhai, M.; Wei, B.; Lyu, B.; Liu, L. Self-Healing and Recyclable Castor Oil-Based Epoxy Vitrimer Based on Dual Dynamic Bonds of Disulfide and Ester Bonds. *ACS Appl. Polym. Mater.* **2024**, *6*, 8399–8408. https://doi.org/10.1021/acsapm.4c01208.

Disclaimer/Publisher's Note: The statements, opinions and data contained in all publications are solely those of the individual author(s) and contributor(s) and not of MDPI and/or the editor(s). MDPI and/or the editor(s) disclaim responsibility for any injury to people or property resulting from any ideas, methods, instructions or products referred to in the content.
